# The Role of 14-3-3ε Interaction with Phosphorylated Cdc25B at Its Ser321 in the Release of the Mouse Oocyte from Prophase I Arrest

**DOI:** 10.1371/journal.pone.0053633

**Published:** 2013-01-10

**Authors:** Jun Meng, Cheng Cui, Yanchun Liu, Minglin Jin, Didi Wu, Chao Liu, Enhua Wang, Bingzhi Yu

**Affiliations:** 1 Department of Biochemical and Molecular Biology, China Medical University, Shenyang, Liaoning Province, China; 2 Center of Clinical Laboratory, Affiliated Hospital, Inner Mongolia Medical University, Hohhot, Inner Mongolia, China; 3 Institute of Pathology and Pathophysiology, China Medical University, Shenyang, Liaoning Province, China; Instituto Gulbenkian de Ciência, Portugal

## Abstract

The protein kinase A (PKA)/Cdc25B pathway plays a critical role in maintaining meiotic arrest in mouse oocytes. However, the molecular mechanism underlying this interchange is not known. In this study, we assessed the role of 14-3-3ε interaction with phosphorylated Cdc25B at its Ser321 as the mouse oocyte is released from prophase I arrest. The 14-3-3ε isoform is a highly conserved protein with various regulatory roles, including maintenance of meiotic arrest. Cdc25B phosphatase is also a key cell cycle regulator. 14-3-3ε binds to Cdc25B-WT, which was abrogated when Ser321 of Cdc25B was mutated to Ala. In addition, we found that 14-3-3ε and Cdc25B were co-localized. Cdc25B was translocated from the cytoplasm to the nucleus shortly before germinal vesicle breakdown (GVBD) during the primary oocyte stage of oogenesis. However, mutation of Ser321 to Ala completely abolished the cytoplasmic localization of Cdc25B. Furthermore, oocytes co-expressing of Cdc25B-WT or Cdc25B-Ser321D and 14-3-3ε were unable to undergo GVBD. In contrast, co-expression of 14-3-3ε and Cdc25B-Ser321A induced GVBD and allowed the process to continue. Down-regulation of 14-3-3ε caused partial meiotic resumption. Taken together, these data indicate that Ser321 of Cdc25B is the specific binding site for 14-3-3ε binding, and that 14-3-3ε is the significant factor in Cdc25B regulation during meiotic resumption of GV stage.

## Introduction

Mammalian oocytes are naturally arrested at prophase I, also named the germinal vesicle (GV) stage, until puberty when luteinizing hormone (LH) induces resumption of meiosis of follicle-enclosed oocytes. The first clear and visible marker of meiotic resumption is germinal vesicle break down (GVBD).It denotes transition from the GV stage to GVBD, a process that resembles the G_2_/M transition of the cell cycle. This transition requires activation of Maturation-Promoting Factor (MPF), comprised of a catalytic subunit Cdc2 and a regulatory subunit cyclinB. The Wee1B/Myt1 kinase inhibits Cdc2 by phosphorylation of Thr14 and Tyr15 on Cdc2 and arrests oocytes at prophase I [1], [2]. The activation of the MPF by removal of inhibitory phosphorylation is catalyzed by the dual-specificity phosphatase Cdc25 [1]. Thus, the prophase I to metaphase I transition can be induced by inactivation of Wee1B/Myt1 kinase and/or activation of Cdc25 phosphatase. In mammalian cells there are three homologues of Cdc25; Cdc25A, Cdc25B and Cdc25C, which play key roles in the regulation of mitosis and meiosis [3], [4]. However, Cdc25B plays an essential role during the prophase I to metaphase I transition in mouse oocytes [5]. It has been reported that Cdc25B^−/−^ female mice are sterile due to the permanent arrest at the GV stage in oocytes. Wild-type Cdc25B protein injected as mRNA into Cdc25B^−/−^ oocytes rescues the phenotype and results in MPF activation and meiotic resumption [5]. In contrast, Cdc25C^−/−^ female mice are fully fertile, meaning that Cdc25C is not essential or has a distinct function in murine meiosis [6]. Cdc25A is exclusively localized in the nucleus at the GV stage. In contrast, Cdc25B is localized in the cytoplasm during the GV stage and translocates into the nucleus shortly before GVBD [7]–[10]. RNAi targeting of Cdc25A in mouse oocytes shows that Cdc25A may not be critical to induce GVBD [10]. These data suggest that Cdc25B is a more potent MPF phosphatase, and is essential for the resumption of meiosis in mouse oocytes versus Cdc25A and Cdc25C.

The 14-3-3 proteins are a family of highly conserved acidic regulatory proteins [11]. They are expressed in all eukaryotic cells from yeast to mammals. Seven isoforms have been identified in mammals, including 14-3-3β, γ, ε, η, σ, τ and ζ. Proteomic and biochemical studies have demonstrated that more than 200 proteins associate with 14-3-3 proteins in vivo [12]–[14]. Among these are proteins involved in cell cycle control, signal transduction, trafficking, apoptosis, stress response, and malignant transformation [15], [16]. Binding of 14-3-3 proteins to specific phospho-serine/threonine motifs in target proteins can variably modify the function of the target proteins, or alter their subcellular localization. Of particular interest is 14-3-3 protein, which has a role in cell-cycle regulation. For instance, 14-3-3 controls the localization of Cdc25B in somatic cells [17]. Studies in Xenopus oocytes have shown that PKA phosphorylates Ser287 of Cdc25C, whereas the heat-stable inhibitor of PKA induces Ser287 dephosphorylation. Phosphorylation of Cdc25C on Ser287 creates a binding site for 14-3-3 adaptor protein, which functionally inactivates Cdc25C through cytoplasmic sequestration and consequential arrest at prophase I of meiosis in Xenopus oocytes [18]–[20].

Our previous studies demonstrated that the Ser321 of Cdc25B (corresponding to Cdc25B-Ser323 of human and Cdc25-Ser287 of Xenopus oocytes) plays a critical regulatory role in the prophase I to metaphase I transition in mouse oocytes, and that phosphorylated Ser321 of Cdc25B is expressed exclusively in the cytoplasm at the GV stage, which could not, however, be detected in GVBD oocytes [9]. These studies suggest that Ser321 of Cdc25B is the potential target of PKA, and that binding of 14-3-3 protein to phosphorylated Cdc25B at its Ser321 induces Cdc25B to relocalize from the nucleus to the cytoplasm. Our recent study demonstrated through LC-MS/MS analysis in vitro that PKA can phosphorylate Ser321 of Cdc25B [21]. To our knowledge, the role and isoforms of 14-3-3 protein in the prophase I stage of meiosis in mouse oocytes have not been examined. In this study, we found that the 14-3-3ε isoform co-localizes with Cdc25B in the cytoplasm at the GV stage in mouse oocytes. Ser321A mutant protein on Cdc25B is mainly localized in the nucleus. However, knockdown of the 14-3-3ε isoform results in partial meiotic resumption. In HEK293 cells, wild-type Cdc25B protein and the phosphomimic Cdc25B-Ser321D mutant can strongly bind to 14-3-3ε whereas binding of the Cdc25B-Ser321A mutant to 14-3-3ε is abrogated. These data suggested that binding 14-3-3ε to Cdc25B via phosphorylated Ser321 mediated by PKA sequesters Cdc25B in the cytoplasm, and that the binding contributes to maintaining prophase I arrest in the mouse oocyte.

## Results

### Identification of 14-3-3 isoforms expressed in GV and GVBD mouse oocytes

**Figure 1:**
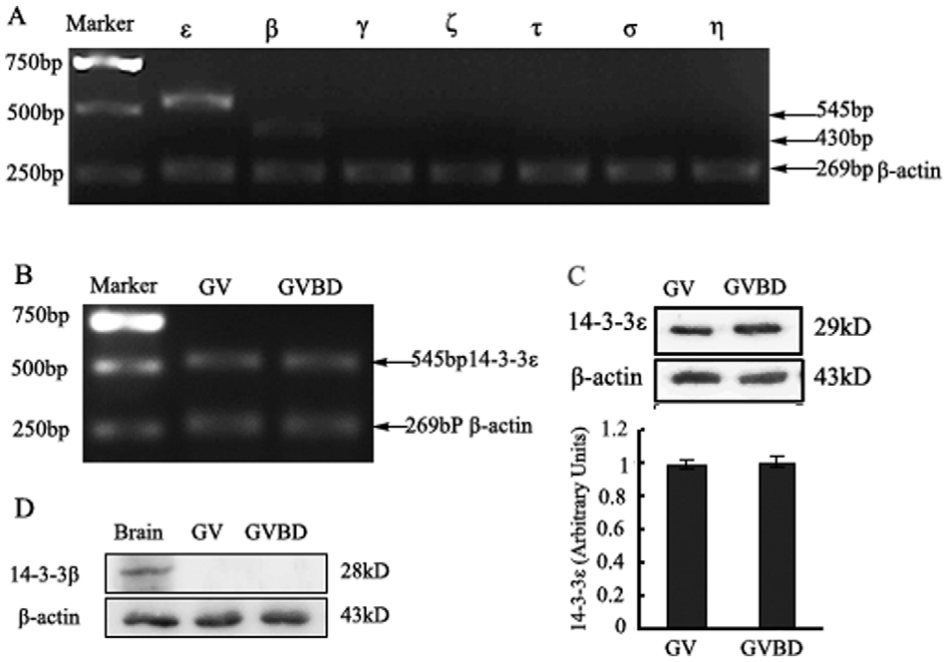
Identification of 14-3-3 isoforms expressed in GV and GVBD mouse oocytes. (A) the mRNA levels of 14-3-3 isoforms at GV stage of mouse oocytes. mRNA of 120 mouse oocytes of GV stage were extracted (described in the“Materials and Methods” Section). RT-PCR products using primers for specific 14-3-3 isoforms are observed in ethidium bromide-stained agarose gel. ε, β, γ, η, σ, τ and ζ represent seven 14-3-3 isoforms. (B) the mRNA levels of 14-3-3ε at GV and GVBD stage of mouse oocytes. Line GV: mouse oocytes at GV stage. Line GVBD: mouse oocytes at GVBD stage. (C) A protein band of approximately 28 kDa from 200 mouse oocytes at GV or GVBD stage is detectable by Western blot with an anti-14-3-3ε antibody (upper panel). In contrast an anti-β-actin antibody detected a low molecular weight band of approximately 43 kDa (lower panel). (D) Western blot analysis 14-3-3β protein expression with an anti-14-3-3β antibody from 300 mouse oocytes at GV or GVBD stage, A protein band of approximately 29 kDa (upper panel). Line Brain: mouse brain protein as positive control. Shown is a representative of three independent experiments.

In order to determine the **e**xpression profile of 14-3-3 isoforms in GV mouse oocytes, RT-PCR was used to amplify the mRNA of 14-3-3β, γ, ε, η, σ, τ and ζ in GV oocytes. The results indicate that only 14-3-3β and 14-3-3ε exist in GV oocytes (Fig. 1A). Transcript identity was confirmed in all cases by DNA sequencing (data not shown). The expression levels of 14-3-3ε mRNA remained unchanged in GV and GVBD oocytes (Fig. 1B). We examined the protein expression of 14-3-3ε and 14-3-3β by Western blot demonstrating that 14-3-3ε was present at constant levels during GV and GVBD stages (Fig. 1C). However, endogenous 14-3-3β was not observed during GV and GVBD stages (Fig. 1D).

### Down-regulation of 14-3-3ε causes partial meiotic resumption of mouse oocytes

**Figure 2:**
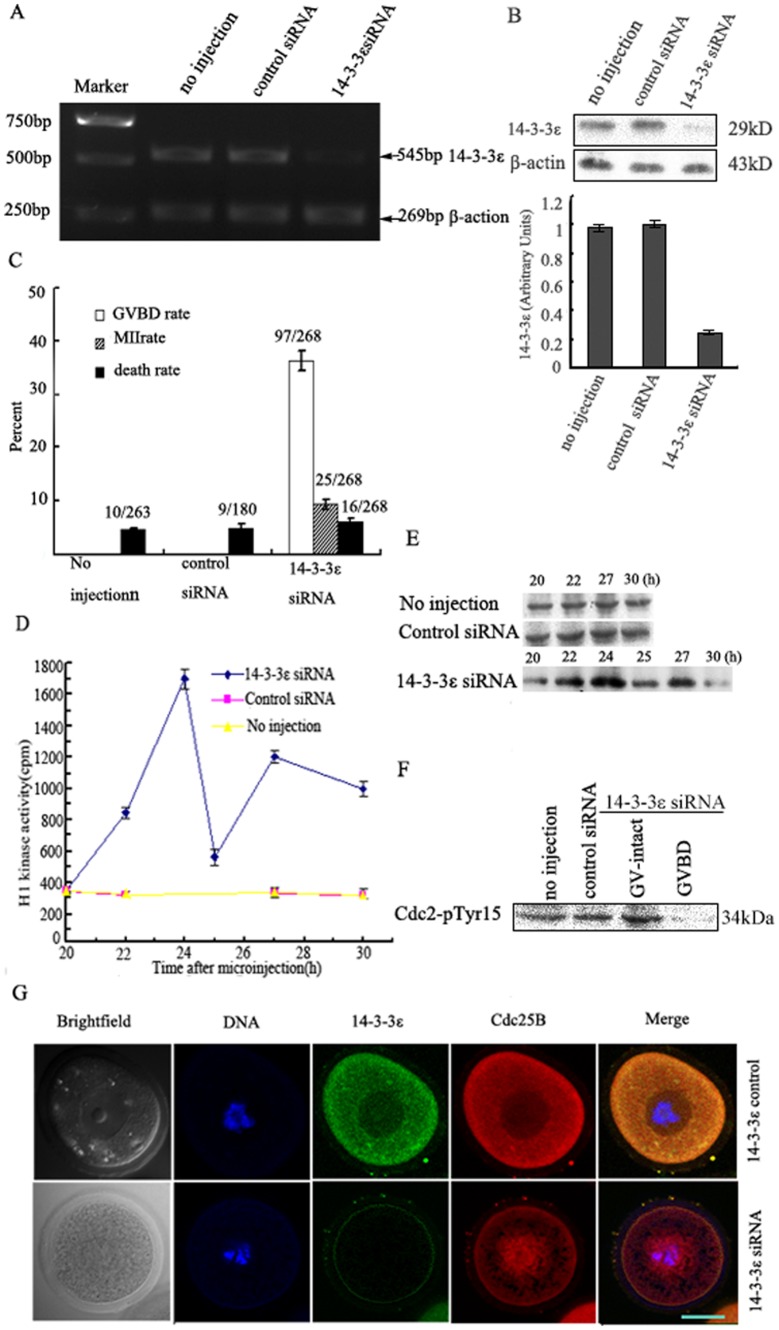
Knockdown of 14-3-3ε by siRNA causes partial meiotic resumption mouse oocytes. (A) 120 GV-oocytes microinjected with 14-3-3ε siRNA or control siRNA (5pl of 20 µM) were collected 24 h after microinjection. Oocytes microinjected with control siRNA and no injection served as control. mRNA of 14-3-3ε was detected by RT-PCR using primers specific for 14-3-3ε. (B) Protein expression of 14-3-3ε was examined by western blot with an anti-14-3-3ε antibody. β-actin used as endogenous internal reference demonstrate 14-3-3ε siRNA specificity and equal loading. (C) at the indicated times, the percentages of germinal vesicle breakdown (GVBD) or MII or death were counted in cultured mouse oocytes after 14-3-3ε siRNA or control siRNA microinjection. GVBD, 24 h; MII or death, 30 h. The total number of oocytes undergoing meiotic maturation or death is given on top of bar graph from three independent experiments. (D) H1 kinase activity in oocytes injected with 14-3-3ε siRNA or control siRNA or no injection groups. Each value was expressed as mean±S.D of at least three independent experiments. (E) Protein extracts from oocytes microinjected with 14-3-3ε siRNA or control siRNA and no injection groups were incubated with histone H1 and [γ^-32^P] ATP. The protein was subjected to sodium dodecyl sulfate-polyacrylamide gel electrophoresis (SDS-PAGE) and incorporation of ^32^P into histone H1 was visualized by autoradiography. (F) Western analysis of phosphorylation status of Cdc2-Tyr15. GV oocytes injected with 14-3-3ε siRNA or control siRNA were collected 24 h after microinjection. The collected oocytes were immunoblotted with anti-pTyr15 of Cdc2 antibody. (G) Co-localization of Endogenous Cdc25B and 14-3-3ε in mouse oocytes injected with 14-3-3ε siRNA or control siRNA. red fluorescent Cdc25B signals and green fluorescent 14-3-3ε signals were co-localized in the cytoplasm in oocytes injected with control siRNA, while red fluorescent Cdc25B signals were translocated from the cytoplasm to the nucleus in oocytes injected with14-3-3ε siRNA. Scale bar = 20 µm.

To explore the role of 14-3-3ε in prophase I-arrested mouse oocytes, a small interference RNA (14-3-3ε siRNA) was microinjected into the cytoplasm of GV-stage oocytes to knock down endogenous 14-3-3ε. The knockdown effect of 14-3-3ε siRNA was evaluated by RT-PCR (Fig. 2A) and Western blot analysis (Fig. 2B). Oocytes injected with control siRNA of low GC and uninjected oocytes were used as negative controls. The results showed that 14-3-3ε siRNA microinjection caused 60–75% depletion of 14-3-3ε (Fig. 2B).

According to the Xenopus oocyte model of the role of 14-3-3ε in prophase I arrest, we postulated that if 14-3-3ε cannot bind to Cdc25B, mouse oocytes would reenter meiosis at the GV stage because Cdc25B would be free to activate the MPF. To test this, we designed 14-3-3ε siRNA. The result showed that 36% the oocytes underwent GVBD 24 h after microinjection of 14-3-3ε siRNA (P<0.01), whereas none of the control-treated oocytes underwent GVBD (Fig. 2C). To investigate a potential mechanism by which 14-3-3ε siRNA regulates meiotic resumption of mouse oocytes at the GV stage, MPF activity was measured at different time points after microinjection of 14-3-3ε siRNA. The results showed that the peak of MPF activity in the14-3-3ε siRNA group was seen 24 h after microinjection of 14-3-3ε siRNA. In contrast, the activity of the MPF remained at low levels in control siRNA and uninjected groups (Fig. 2D, E). Cdc2-Tyr15 was dephosphorylated in the GVBD oocytes injected with 14-3-3ε siRNA 24 h after injection, but remained phosphorylated at the same time point in GV-intact oocytes injected with 14-3-3ε siRNA, control siRNA, and uninjected oocytes, Cdc2-Tyr15 dephosphorylation in the RNAi-treated fraction undergoing GVBD differed significantly from controls 24 h after injection (Fig. 2F). These results were consistent with the MPF activity measurements. We discovered that the uninjected oocytes or those injected with control siRNA were blocked in the prophase I stage of meiosis. In contrast, those injected with 14-3-3ε siRNA partially underwent GVBD. We also used indirect immunofluorescence to determine the location of endogenous Cdc25B and 14-3-3ε in mouse oocytes injected with 14-3-3ε siRNA or control siRNA. As shown in Fig. 2G, red fluorescent Cdc25B signals and green fluorescent 14-3-3ε signals were co-localized in the cytoplasm in oocytes injected with control siRNA, while red fluorescent Cdc25B signals were translocated from the cytoplasm to the nucleus in oocytes injected with14-3-3ε siRNA. These data suggest that 14-3-3ε may be involved in prophase I arrest in mouse oocytes and that 14-3-3ε may influence the intracellular localization of Cdc25B.

### Ser321 of Cdc25B is the specific binding site for 14-3-3ε

**Figure 3:**
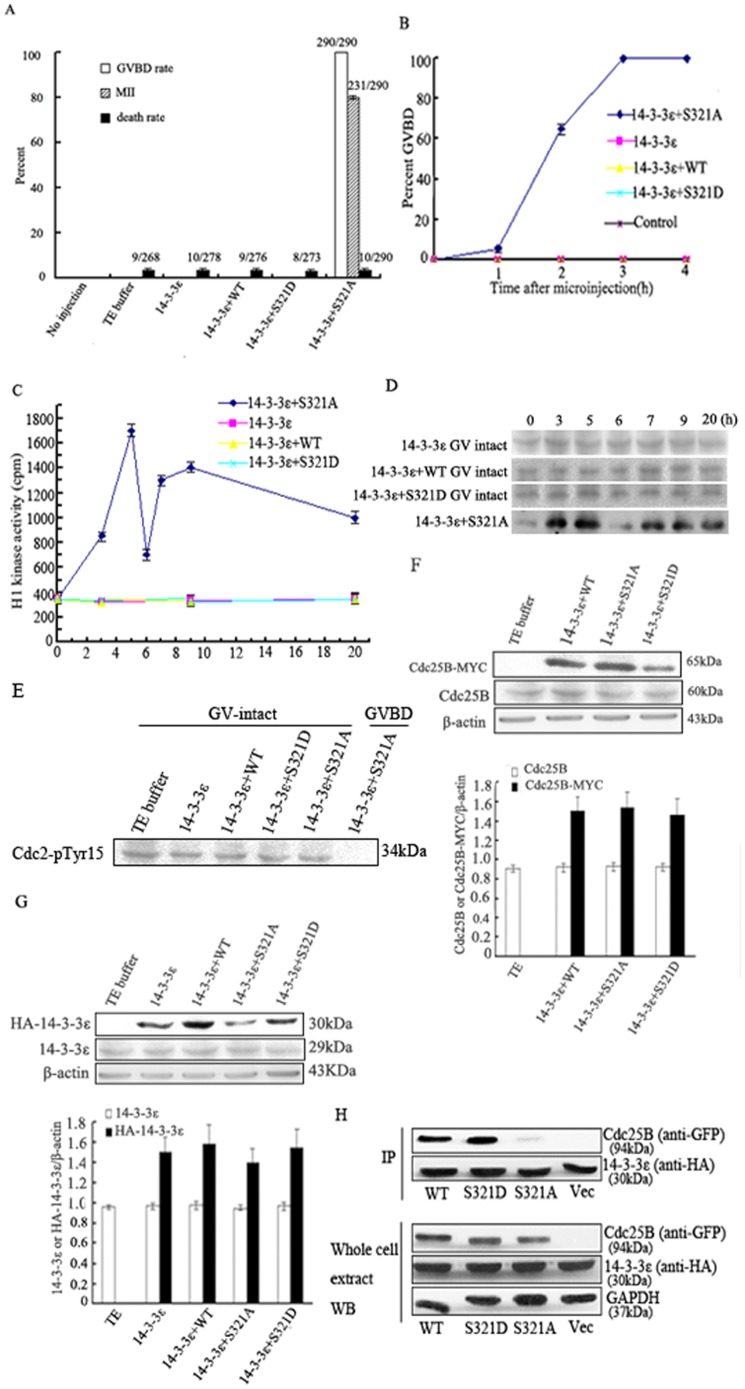
Ser321 of Cdc25B is the sole site responsible for 14-3-3ε binding. Effects of microinjection with HA-tagged-14-3-3ε mRNA solely or co-injection with MYC-tagged-Cdc25B-WT mRNA, MYC-tagged-Cdc25B-Ser321A mRNA or MYC-tagged-Cdc25B-Ser321D mRNA and HA-tagged- 14-3-3ε mRNA on meiotic resumption of mouse oocytes. Experiments were performed in the continued presence of dbcAMP(A–G). HEK293 cells were transfected with 14-3-3ε together with empty vector, Cdc25B-WT , Cdc25B-Ser321A or Cdc25B-Ser321D (H). (A) at the indicated times, the percentages of germinal vesicle breakdown (GVBD) or MII or death were counted in cultured mouse oocytes after various mRNAs microinjection. GVBD, 3 h; MII or death, 20 h. The total number of oocytes undergoing meiotic maturation or death is given on top of bar graph from three independent experiments. (B) the GVBD rate at indicated time points in cultured mouse oocytes after various mRNAs microinjection. (C) H1 kinase activity in oocytes injected with various mRNAs. Each value was expressed as mean±S.D of at least thee independent experiments. (D) Protein extracts from oocytes microinjected with various mRNAs were incubated with histone H1 and [γ^−32^P] ATP. The protein was subjected to sodium dodecyl sulfate-polyacrylamide gel electrophoresis (SDS-PAGE) and incorporation of ^32^P into histone H1 was visualized by autoradiography. (E) Western analysis of phosphorylation status of Cdc2-Tyr15. GV oocytes injected with various mRNAs were collected 2 h after microinjection. The collected oocytes were immunoblotted with anti-pTyr15 of Cdc2 antibody. (F) Western analysis of Cdc25B-MYC and endogenous Cdc25B expression. The oocytes co-injected with Cdc25B-WT mRNA, Cdc25B-Ser321A mRNA or Cdc25B-Ser321D mRNA and 14-3-3ε mRNA were collected 3 h after injection and their proteins were immunoblotted with anti-myc, anti-Cdc25B or anti-beta-actin antibody. Bars represent means±S.D of thee independent experiments. (G) Western analysis of HA-14-3-3ε and endogenous 14-3-3ε expression. The oocytes injected with 14-3-3ε mRNA solely or co-injected with Cdc25B-WT mRNA, Cdc25B-Ser321A mRNA or Cdc25B-Ser321D mRNA and 14-3-3ε mRNA were collected 3 h after injection and their proteins were immunoblotted with anti-HA, anti-14-3-3ε or anti-beta-actin antibody. Bars represent means±S.D of thee independent experiments. (H) Western blot analysis of Cdc25B interaction with 14-3-3ε (IP) and Western blot analysis demonstrating the expression of HA-14-3-3ε and EGFP-Cdc25B in the cell lysates used to immunoprecipitate Cdc25B protein shown in (IP), an anti-GAPDH antibody was used to determine equal loading of the gel.

Our previous study demonstrated that Cdc25B-WT and Cdc25B-Ser321A can bypass the inhibitory effect of PKA on meiotic resumption. The Cdc25B-Ser321A mutant had a more potent effect on GVBD induction than Cdc25B-WT, while the Cdc25B-Ser321D mutant displayed similar activities to WT protein [9]. To test whether binding 14-3-3ε to Ser321 of Cdc25B blocked meiotic resumption, oocytes were first incubated in MB Medium containing 200 µM dibutyryl cAMP (dbcAMP) and 3 h later microinjected with HA-tagged-14-3-3ε mRNA solely or co-injected with MYC-tagged-Cdc25B-WT mRNA, MYC-tagged-Cdc25B-Ser321A mRNA or MYC-tagged-Cdc25B-Ser321D mRNA at a concentration of 500 µg/ml. After microinjection the oocytes were transferred back into the MB Medium containing 200 µM dbcAMP. Twenty hours after microinjection of various mRNAs, the rate of GVBD in oocytes co-injected with 14-3-3ε mRNA and Cdc25B-Ser321A mRNA group was 100% (with 5% at 1 h, 65% at 2 h, and 100% at 3 h) and 79% progressed to MII (P<0.01, Fig. 3A,B). None of the oocytes co-injected with 14-3-3ε mRNA plus Cdc25B-WT or Cdc25B-Ser321D mRNA were able to undergo GVBD until at least 20 h after co-injection. In addition, none of the oocytes injected with 14-3-3ε mRNA or TE buffer alone were able to undergo GVBD until 20 h after microinjection. Fewer than 4% of oocytes were dead after the various injections (p>0.05).

We also measured the MPF activity and phosphorylation status of cdc2-Tyr15 in oocytes injected with various mRNAs. MPF activity in the GVBD oocytes co-injected with 14-3-3ε and Cdc25B-Ser321A mRNAs fluctuated during the maturation process, being low in the GV oocytes and high in the first and second metaphase oocytes, with a transient decrease at first polar body (PB) emission. In contrast, MPF activity in other solely injected or co-injected mRNA groups remained at a stable low level. MPF activity of the oocytes co-injected with 14-3-3ε and Cdc25B-Ser321A mRNAs differed significantly from the other injected groups (Fig. 3C,D, p<0.01). Cdc2-Tyr15 was dephosphorylated in the GVBD oocytes co-injected with 14-3-3ε and Cdc25B-Ser321A mRNAs 2 h after co-injection, while being phosphorylated at the same time points in other injected groups of GV-intact oocytes, Cdc2-Tyr15 dephosphorylation in the oocytes fraction undergoing GVBD co-injected with 14-3-3ε and Cdc25B-Ser321A mRNAs differed significantly from the GV-intact oocytes co-injected with 14-3-3ε and Cdc25B-Ser321A mRNAs, and also differed significantly from the GV-intact oocytes injected with other mRNAs 2 h after injection (Fig. 3E). These results were consistent with the MPF activity measurements. As noted, activation of endogenous MPF was inhibited in mouse oocytes co-expressing Cdc25B-WT or Cdc25B-Ser321D and 14-3-3ε, but co-expressing 14-3-3ε had hardly any effect on the activity of the Ser321A mutant protein. These data suggested that 14-3-3ε binding to phosphorylatable Ser321 blocked MPF activity via Cdc25B inhibition. The over-expression of 14-3-3ε had no apparent direct effect on MPF activity or activation, namely, the over-expression of 14-3-3ε did not affect prophase I to metaphase I transition in mouse oocytes. Moreover, various Cdc25B-MYC fusion proteins and HA-14-3-3ε fusion protein at the protein levels were probed by Western blot in various mRNA-injected oocytes 3 h after microinjection (Fig. 3F, G). These results indicated that all injected mRNAs were translated efficiently and exogenously expressed protein levels were higher than endogenously expressed protein levels (p<0.01).

To determine whether 14-3-3ε can bind Cdc25B at Ser321, HEK293 cells were transfected with HA-14-3-3ε together with empty vector, EGFP-Cdc25B-WT, EGFP-Cdc25B-Ser321A or EGFP-Cdc25B-Ser321D. Recombinant protein expression was confirmed in lysates from transfected cells (Fig. 3H-Lysate). 14-3-3ε was immunoprecipitated with anti-HA beads followed by Western blotting to detect Cdc25B binding with 14-3-3ε. In contrast to Cdc25B-Ser321A, wild-type Cdc25B and Cdc25B-Ser321D were found in complex with 14-3-3ε (Fig. 3H), indicating that 14-3-3ε physically interacts with phosphorylated Cdc25B at Ser321, but not with unphosphorylated Cdc25B.

### Co-localization of endogenous Cdc25B and 14-3-3ε

**Figure 4:**
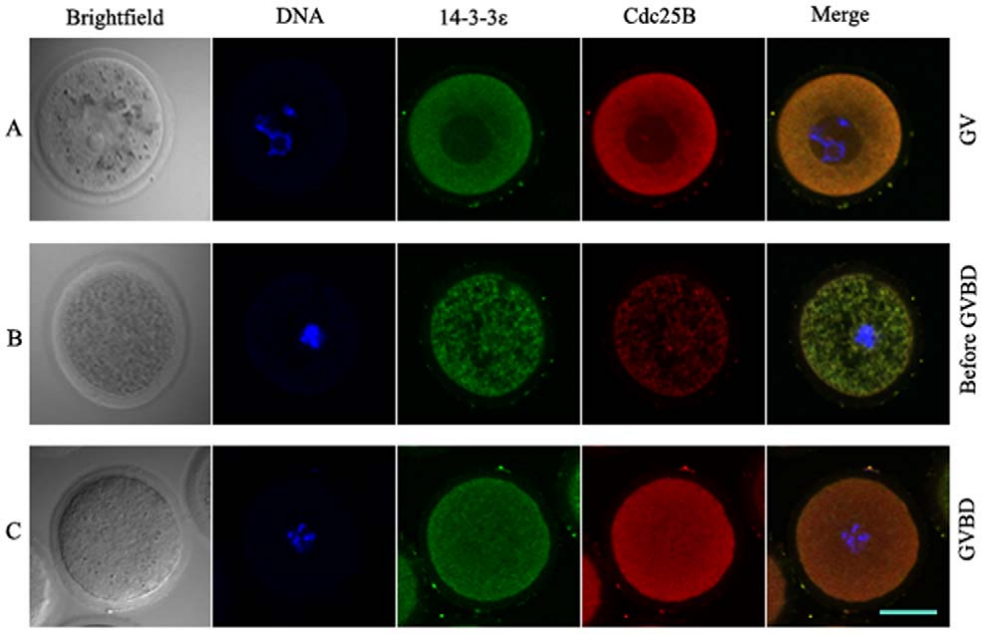
Co-localization of endogenous Cdc25B and 14-3-3ε. (A) red fluorescent Cdc25B signals and green fluorescent 14-3-3ε signals were colocalized in the cytoplasm in GV-stage oocytes. (B) the partial red fluorescent signals were translocated to the nucleus in oocytes before GVBD (C) after GVBD, the red fluorescent signals and the green fluorescent signals were evenly distributed in the whole cell. Scale bar = 20 µm.

Using indirect immunofluorescence, we observed the co-localization of endogenous Cdc25B and 14-3-3ε in mouse oocytes. As shown in Fig. 4, red fluorescent Cdc25B signals and green fluorescent 14-3-3ε signals were co-localized in the cytoplasm in GV-stage oocytes (Fig. 4A). The partial red fluorescent Cdc25B signals were translocated to the nucleus in oocytes before GVBD (Fig. 4B). However, after GVBD the red fluorescent signals and the green fluorescent signals were evenly distributed in the whole cell (Fig. 4C). These results indicated that the shuttling of Cdc25B is accompanied with meiotic resumption.

### Co-localization of exogenously expressed Cdc25B and 14-3-3ε

**Figure 5:**
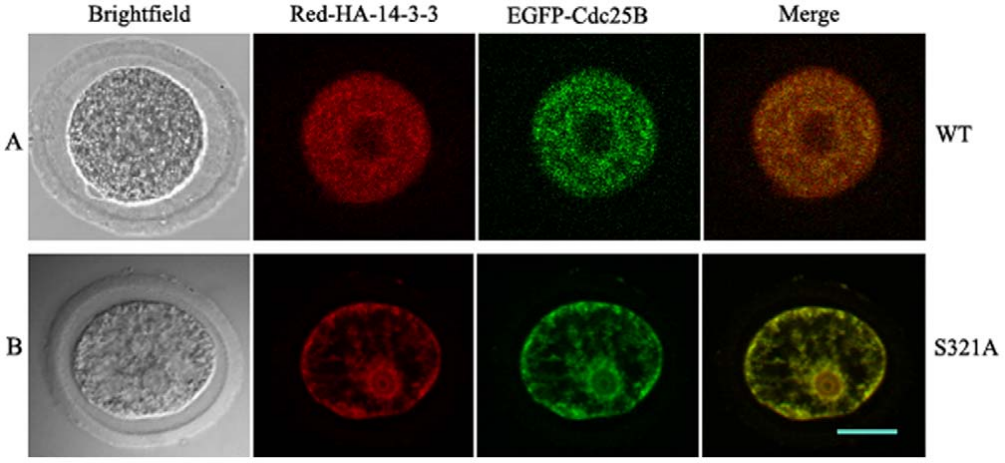
Co-localization of exogenously expressed Cdc25B and 14-3-3ε. (A) red fluorescent 14-3-3ε signals and green fluorescent Cdc25B-WT signals were co-localized in the cytoplasm in GV-stage oocytes. (B) red fluorescent 14-3-3ε signals and green fluorescent Cdc25B-Ser321A signals were distributed in the whole cell, but particularly dense in the nucleus. Scale bar = 20 µm.

To test the effect of the Ser321 phosphorylation on subcellular localization of Cdc25B and 14-3-3ε, pEGFP-Cdc25B-WT or pEGFP-Cdc25B-Ser321A was co-injected with pRFP-HA-14-3-3ε into GV-stage oocytes, and the injected oocytes were transferred into MB medium containing 200 µM dbcAMP to allow protein expression. As shown in Fig. 5, green fluorescent Cdc25B signals and red fluorescent 14-3-3ε signals were co-localized in the cytoplasm of mouse oocytes at the GV stage (Fig. 5A). In contrast, green fluorescent Cdc25B-S321A signals and red fluorescent 14-3-3ε signals were distributed in the whole cell, but were particularly dense in the nucleus (Fig. 5B). Moreover, localization was independent of the amount of recombinant protein expressed and of the tag used to detect the expressed proteins [8]. These results confirm that Ser321 of Cdc25B was indeed critical for the subcellular localization of Cdc25B.

## Discussion

PKA has been documented as a pivotal regulator in meiosis and mitosis arrest [22]–[27]. Our previous studies have established that PKA acts as negative regulator of MPF and that Ser321 of Cdc25B plays a critical regulatory role in meiotic resumption as well as in the development of mouse one-cell embryos by modification of phosphorylation and dephosphorylation [9], [28], [29]. Meanwhile, we have confirmed that Ser321 of Cdc25B was phosphorylated in the GV stage, and dephosphorylated during GVBD [9]. Recently, our results from LC-MS/MS analysis demonstrated that PKA can phosphorylate the Ser149, Ser229, and Ser321 sites of Cdc25B, corresponding to the phosphorylation sites predicted by the Scansite software [21]. Therefore, Cdc25B is identified as the direct downstream substrate of PKA in mammalian cells. These data strongly suggest that at high cAMP levels, PKA is activated and phosphorylates Cdc25B on Ser321. The phosphorylation of Cdc25B in turn inhibits Cdc25B activity, thereby maintaining prophase I arrest of oocytes. It has been shown that prophase I arrest in Xenopus oocytes depends on phosphorylation of Ser287 of Cdc25 mediated by PKA and Cdc25 binding by 14-3-3ε protein contributes to the maintenance of prophase I arrest in the oocytes [19], [20]. We explored whether prophase I-arrested mouse oocytes express different 14-3-3 isoforms . Our study showed that the 14-3-3ε isoform was present at constant levels in GV and GBVD oocytes. Although 14-3-3β mRNA was detected by RT-PCR, 14-3-3β protein was not detected by Western blot because of very low expression. 14-3-3γ, σ, ζ, τ and η isoforms were not detected at the mRNA level by RT-PCR for GV oocytes or for GVBD oocytes (unpublished data). Therefore, we will focus our discussion on the effect of 14-3-3ε on prophase I arrest in the mouse oocytes. In this study, we provide experimental evidence demonstrating the interaction and co-localization of 14-3-3ε and Cdc25B in mouse oocytes.

14-3-3 proteins are a family of abundant, widely expressed acidic polypeptides. They are expressed in all eukaryotic cells and are highly conserved in protein sequences and functions in organisms ranging from yeast to mammals [30]. The 14-3-3 proteins bind to target proteins containing specific phospho-serine/theonine motifs, which can in turn alter their catalytic activity or cellular localization [31]–[34]. Our immunofluoresence experiments revealed a restricted cytoplasmic co-localization of 14-3-3ε and Cdc25B in prophase I-arrested oocytes and nuclear localization of Cdc25B shortly before GVBD. These results are in agreement with previous reports [8], [9]. Interestingly, some 14-3-3ε transferred from the cytoplasm to the nucleus. This was accompanied by the translocation of Cdc25B from the cytoplasm to the nucleus. The majority of 14-3-3 molecules are present in the cytoplasm and in the absence of bound ligands, 14-3-3 homes to the nucleus [35]. Another report demonstrated that 14-3-3 normally transits into and out of the nucleus [36]. Therefore, it is possible that in the absence of bound ligands of Cdc25B some 14-3-3ε is localized to the nucleus, as in Fig. 4 and 5. Emerging evidence suggests that 14-3-3 proteins are key regulators of the cell cycle. Our previous study demonstrated that overexpression of Cdc25B-Ser321A alone in oocytes can induce Cdc2-Tyr15 dephosphorylation and meiotic resumption much more efficiently than overexpression of Cdc25B-WT or overexpression of Cdc25B-Ser321D [9], whereas mutation at the Ser149 or Ser229 sites of Cdc25B has no effect on meiotic resumption (Yu, unpublished data). These results suggest that 14-3-3 may bind to Ser321 and inhibit Cdc25B activity, which then fails to activate MPF. Here our findings demonstrated that co-expression of 14-3-3ε and Cdc25B-Ser321A in oocytes was able to induce GVBD, whereas co-expression of Cdc25B-WT or Cdc25B-Ser321D and 14-3-3ε was unable to induce GVBD. In addition, overexpression of 14-3-3ε alone did not affect the prophase I to metaphase I transition. Thus, the ability of the co-expression of 14-3-3ε to inhibit Cdc25B-dependent activation of MPF is likely related to 14-3-3 binding of wild-type Cdc25B, which is defective in the Ser321A mutant. These findings further consolidated our conclusion that 14-3-3ε binds to phosphorylated Ser321 and inhibits Cdc25B activity. Accumulated anecdotal evidence indicates that 14-3-3 negatively controls G_2_/M transition by binding to Cdc25 and providing scaffolding or a cover that hides specific motifs, such as the nuclear localization signal (NLS) or nuclear export signal (NES) [32], [33], [37]–[39]. Our RNA interference results showed that down-regulation of 14-3-3ε resulted in partial meiotic resumption. Release of 14-3-3 proteins binding to phosphorylated Cdc25 during interphase is one of the early steps of the G_2_/M transition [40]. Therefore, it is possible that Ser321 of Cdc25B is phosphorylated by PKA and binds to 14-3-3ε in the cytoplasm at prophase I arrest, whereas Cdc25B performs its onset of resumption of meiosis after the knockdown of 14-3-3ε, thus activating MPF at prophase I arrest in mouse oocytes. 14-3-3ε may control the timing of Cdc25B activation under appropriate conditions and negatively regulate Cdc25B.

Several groups have reported that the binding of 14-3-3, specifically at Ser309 of Cdc25B1 or Ser323 of Cdc25B2 or Cdc25B3, which is the equivalent site to mouse Cdc25B-Ser321, results in the cytoplasmic localization of Cdc25B, reinforcing the theory of its redistribution from the nucleus to the cytoplasm as a critical G_2_/M checkpoint [41]–[43]. Yamashita and coworkers [37] demonstrated that 14-3-3ε binds Cdc25B1 at Ser309 (or Ser323 of Cdc25B2 or Cdc25B3), and controls Cdc25B localization in human somatic cells. In this study, we confirm a similar mechanism in which 14-3-3ε regulates murine Cdc25B though Ser321; 14-3-3ε can bind the wild-type Cdc25B, nevertheless, when Ser321 of Cdc25B is mutated to an unphosphorylated residue Ala, 14-3-3ε binding is abrogated (Fig. 3H). The co-localization of exogenously expressed 14-3-3ε and Cdc25B-WT or Cdc25B-S321A further demonstrates that Ser321 of Cdc25B is critical for the localization of Cdc25B. It suggests that localization of Cdc25B is determined by 14-3-3ε in meiotic maturation. Taken together, these data strongly suggest that Cdc25B-Ser321 phosphorylation may provide a docking site for consequent 14-3-3ε binding, which in turn masks the nuclear localization signal (NLS) of Cdc25B, thereby causing nuclear exclusion of the protein without affecting its phosphatase activity. When Ser321 of Cdc25B is mutated to an unphosphorylated residue Ala, the 14-3-3ε binding is abrogated, and homeostasis of nucleus export and import is subsequently restored.

Based on the literature and our findings, we propose a model for the regulation of Cdc25B in which, at high cAMP levels, PKA can phosphorylate Cdc25B-Ser321 directly. Phosphorylated Ser321 of Cdc25B allows 14-3-3ε to bind Cdc25B, which results in Cdc25B being sequestered in the cytoplasm away from MPF in the nucleus, therefore blocking its activation of MPF. Oocytes are then arrested at prophase I. Our findings further expound the molecular mechanism by which 14-3-3ε-mediated inhibition of Cdc25B activity maintains prophase I arrest in mouse oocytes.

In general, our findings not only further confirm the role of the PKA/Cdc25B-Ser321/14-3-3ε pathway in mouse oocytes, also provide a novel mode of 14-3-3ε regulating Cdc25B activity by subcellular relocalization at the prophase I to metaphase I transition.

## Materials and Methods

### Animal

Kunming genealogy mice were provided by the Department of laboratory Animals, China Medical University (CMU). All experiments were performed at CMU in accordance with NIH Guidelines for the Care and Use of Laboratory Animals. The protocol for animal handling and the treatment procedures were reviewed and approved by the CMU Animal Care and Use Committee. Reagents, unless otherwise specified, were obtained from Sigma (St. Louis, MO).

### Collection and Culture of Mouse Oocytes

GV oocytes were collected from the ovaries of 21-to24-day-old Kunming mice that had been injected with 5 IU of pregnant mare's serum gonadotrophin (PMSG) 46–48 h before collection [44]. Ovaries were placed in M2 medium containing 200 µM dbcAMP to prevent GVBD. The follicles were punctured with a fine needle to release the cumulus-enclosed oocytes or naturally denuded oocytes. Cumulus cells were subsequently removed by repeated pipetting with a mouth-operated micropipette. Denuded oocytes were then placed in drops of media under oil and cultured at 37°C in a 5% CO_2_ atmosphere. Oocytes culture was performed in Waymouth's MB752/1 Media (Invitrogen) supplemented with 100 µg/ml sodium pyruvate, 50 IU/ml penicillin, 50 µg/ml streptomycin sulfate, 3 mg/ml bovine serum albumin (BSA, Fraction V), called MB medium [45].

### Construction of mRNA Expression Vectors

To create HA-tagged 14-3-3ε, the sequence encoding the HA tag was introduced immediately upstream of the initiation start codon of 14-3-3ε cDNA, Forward primer: 5′-ATGTACCCATACGATGTT CCAGATTACGCTGATGATCGGGAGGATCT-3′, reverse primer: 5′-GCTTTTATTTCGTCTCACTGATTC TCATCTT-3′, mRNA was extracted from mouse liver using the Ellustra™ QuickPrep MicromRNA Purification Kit (GE Healthcare UK Limited,UK). The coding sequence of HA-14-3-3ε was amplified by RT-PCR using a one-step RT-PCR kit (Invitrogen). The product was cloned into the mRNA expression vector pcDNA3.1-ZEO (+) (Invitrogen) and named pcDNA3.1-ZEO- HA-14-3-3ε. The construct was sequenced to verify correct insertion.

For analysis of the subcellular localization of 14-3-3ε, the construct was subcloned in-frame into the pmax-RFP-C vector (Clontech) to express fusion proteins of RFP-HA-14-3-3ε.

Other constructs, pcDNA3.1-Myc-Cdc25B-WT, pcDNA3.1-Myc-Cdc25B-Ser321A, pcDNA3.1-Myc- Cdc25B-Ser321D, pEGFP-Cdc25B-WT, pEGFP-Cdc25B-Ser321A, and pEGFP-Cdc25B-Ser321D, were constructed in our Laboratory.

### In Vitro Transcription

As described in our previous report [9], all pcDNA3.1-Myc constructs were linearized with AgeI. pcDNA3.1-ZEO-HA-14-3-3ε was linearized with XbaI and transcribed in vitro into 5′-capped mRNA for microinjection using a mMESSAGE mMACHINE kit (Ambion).

### Microinjection and Morphological Analysis

Various Cdc25B mRNAs or plasmids and HA-Tagged 14-3-3ε mRNA or plasmids were microinjected into the cytoplasm or nucleus of GV-Stage oocytes using a micropipette and Eppendorf manipulators mounted on an Olympus inverted microscope, as in our previous report [9]. Oocytes were placed in a drop dbcAMP-M2 medium under paraffin oil in a lid of a 3 cm Falcon culture dish. The injected oocytes were subsequently scored for the continued presence of the GV, GVBD, PB formation, and death using an inverted microscope fitted with a differential interference contrast (DIC) lens. The typical injection volumes were 5% (10pl, cytoplasm) and 1% (2pl, nucleus).

To observe the subcellular co-localization of various Cdc25B and 14-3-3ε, plasmid DNA of pEGFP-Cdc25B-WT, pEGFP-Cdc25B-Ser321A or pEGFP-Cdc25B-Ser321D were co-injected with pmax-RFP-HA-14-3-3ε into the nucleus of GV oocytes at the concentration of 1 µg/µl. After microinjection the oocytes were further incubated in MB medium containing 200 µM dbcAMP until a distinct fluorescent signal was detected.

### RT-PCR

mRNAs were extracted from mouse oocytes in the GV or GVBD stage using the Ellustra™ QuickPrep MicromRNA Purification Kit (GE Healthcare UK Limited,UK). RT-PCR was performed using RNA PCR Kit (AMV) Ver 3.0 (TakaRa). Each primer was designed according to published mouse cDNA. 14-3-3β (NM_018753.6), forward primer: 5′-GAACGTGGTAGGTGCCCGCC-3′ and reverse primer: 5′-GGCCAGGCTGCAGGCCTTTT-3′, designed for 430 bp. 14-3-3ε (NM_009536.4), forward primer: 5′-GACCGTGCCTGCAGGTTGGC-3′ and reverse primer:5′-CTTGCCAGTGTGGCCGGAGA-3′, designed for 545 bp. 14-3-3η (NM_011738.2), forward primer: 5′-GATATGGCCTCCGCCATGAAGGC- G-3′ and reverse primer: 5′-CATCCTGCTGGTCGCTCGTCCAGAG-3′, designed for 655 bp. 14-3-3τ (NM_011739.3), forward primer: 5′-GTCTGACGCGCTCTCTCCTCGCT-3′ and reverse primer: 5′-GGCGGATTGGATGCGTAGGCTG-3′, designed for 615 bp. 14-3-3γ (NM _018871.3), forward primer: 5′-TCCCTCCGACACACGAGCTCCAA-3′ and reverse primer: 5′-TGGCCACTTCTGCCAGGTAACG-3′, designed for 520 bp. 14-3-3ζ (NM_011740.3), forward primer: 5′-GTGTCTGCGGAGCGGCTGTAGC-3′ and reverse primer: 5′-TCTGGTTGCGAAGCATTGGGGA-3′, designed for 484 bp. 14-3-3σ (NM_018754.2), forward primer:5′-CAGTTCGCCCGTCTGTCTGTCCA-3′ and reverse primer: 5′-GATGGGGTTGGTAGGCGGCATC-3′, designed for 538 bp. β-actin (NM_007393.3), forward primer: 5′-CGGTCCACACCCGCCACC-3′ and reverse primer: 5′-GTCGTCCCAGTTGGTAACAATGCC-3′, designed for 269 bp. All of the primers were designed to span the introns of the rodent gene. The RT reaction was carried out for one cycle at 50°C for 30 min; 99°C for 5 min; 5°C for 5 min. Aliquots of 5 µl of first-strand cDNA were mixed with 20 µl of the PCR mixture. The PCR reaction was carried out in three steps as follows: 94°C for 2 min (one cycle); 94°C for 30 sec,52°C for 30 sec, and 72°C for 1 min (32 cycle); 72°C for 10 min (one cycle). The PCR products were analyzed by electrophoresis on 1.5% agarose gel stained with ethidium bromide to visualize PCR products on a UV transilluminator.

### Immunofluorescence

Oocytes at the GV or G2/M transition or in the GVBD stage were washed in PBS with 0.1% BSA, and fixed in 4% paraformaldehyde in PBS (pH 7.4) for 1 h at room temperature. After being permeablilzed with 0.1% TritonX-100 in PBS at room temperature for 30 min, oocytes were blocked in 5% BSA in PBS for 1 h and incubated overnight at 4°C with polyclonal goat anti-Cdc25B antibody diluted 1∶100(Santa Cruz Biotechnology, Santa Cruz,CA) and polyclonal rabbit anti-14-3-3ε antibody diluted 1∶800 (Abcam) at 4°C . After being washed three times in PBS with 0.1% BSA, the oocytes were incubated with FITC- conjugated goat anti-rabbit secondary antibody (dilution1∶50 in PBS) and TRITC-conjugated rabbit anti-goat secondary antibody (dilution 1∶50 in PBS) at 37°C for 1 h. Then, The DNA was stained with 25 µg/ml Hoechst33258 for 10 min at RT. The signals denoting subcellular localization of endogenous Cdc25B and 14-3-3ε staining were detected by a Laser Confocal Scanning Microscope at 488 nm and530 nm and 260 nm, respectively.

### Western blotting

Oocytes at the indicated times were lysed, subjected to SDS-PAGE (12%), and transferred to nitrocellulose membranes. Blots were blocked for 1 h at room temperature in 5% milk in Tris-buffered saline(TBS) containing 0.05% Tween20 (TBST). The blots were probed overnight at 4°C in 1% milk in TBST with the following antibodies: rabbit anti-14-3-3ε (1∶1000) (Abcam); rabbit anti-14-3-3β (1∶800) (cellsignaling); rabbit anti-Myc (1∶1000) (Clontech); rabbit anti-HA (1∶800) (Sigma); Tyr(P)15 of Cdc2 (1∶500; Santa Cruz Biotechnology) and rabbit anti-β-actin (1∶400) (Santa Cruz Biotechnology). The HRP-conjugated anti-rabbit or anti goat secondary antibody at a dilution of 1∶5000 was used for detection (Beijing Zhongshan Biotechnology, China). Proteins were visualized using the enhanced chemiluminescence (ECL) detection system (Pierce Biotechnology). The proteins expression of 14-3-3ε and β-actin were detected by Western blot. Densitometry of bands was performed with Quantity One Software, densitometry of 14-3-3ε bands/densitometry of β-actin bands was used as quantitation of endogenous 14-3-3ε expression. Bars represent means ±S.D of three independent experiments.

### Assay of MPF Activity

MPF kinase activity was measured by a histone H1 kinase assay [46]. The kinase assay was performed according to a similar procedure, as described in our previous report [9].

### Microinjection of Stealth siRNA in mouse oocytes

For targeted knockdown of 14-3-3ε, three Stealth siRNAs for mouse 14-3-3ε were purchased from Invitrogen. Each Stealth siRNA is a 25 bp duplex oligoribonucleotide. We tested three Stealth siRNA oligoribonucleotides and selected the most effective duplex (siRNA1) for our experiments. The sequence of 14-3-3ε siRNA1 was 5′-UGUACAUCCAGAAUGUCACAACAGA-3′. A 25 bp duplex oligoribonucleotide of Stealth RNAi Neg Ctl LO GC (Invitrogen) was used as the control Stealth siRNA. All Stealth siRNA duplexes were resuspended in 1 ml DEPC-treated water according to the manufacturer's instructions and stored in single-use aliquots at −20°C. GV-stage oocytes were transferred to M2 medium containing 180 µM dbcAMP, and the oocyte cytoplasm was injected with 5 pl of siRNA(20 µM) solution. Following microinjection, oocytes were transferred to MB medium containing 180 µM dbcAMP and incubated at 37°C in 5% CO_2_ for 24 h, and GVBD was scored with a DMI4000B inverted microscope fitted with a differential interference contrast (DIC) lens.

### Cell culture and transfection

HEK293 cells (ATCC number CRL-1573) were cultured in Dulbecco's Modified Eagle's medium (DEME) (Sigma) supplemented with 10% fetal bovine serum (FBS) (Invitrogen)), 100 units/ml penicillin and 10 µg/ml streptomycin. Transient transfections were performed with FuGENE6 (Roche Molecular Biochemicals, Germany) according to the manufacturer's instructions. For immunoprecipitation, cells were typically seeded at 2.0×10^6^ per well. After sitting overnight, cells were co-transfected with 5 µg of EGFP- tagged Cdc25B (WT, Ser321A, Ser321D) or EGFP-C3 Vector and 5 µg of RFP-HA-14-3-3ε DNA. Transfected cells were processed for immunoblotting or immunoprecipitation after 48 h.

### Preparation of crude cells extracts, immunoprecipitation and immunoblotting

Transfected cells were lysed in immunoprecipitation (IP) buffer (50 mM Tris-HCl, 0.25%w/v Deoxycholate, 1%NP40, 150 mM NaCl, 1 mM EDTA, pH 7.4) supplemented with a protease inhibitor mix and a phosphatase inhibitor mix. The protease inhibitor mix contained 5 µg/ml leupeptin, pepstatin A, aprotinin, 1 mM PMSF. The phosphatase inhibitor mix consisted of a 1∶100 dilution of Phosphatase inhibitor cocktail II (Sigma, USA), 25 mM NaF, 0.1 mM sodium orthovanadate and 25 mM β-glycerophosphate. Cell lysates were incubated with mouse monoclonal anti-HA antibody (Covance) and protein G-agarose beads (GE Healthcare). The precipitates were then washed with a lysis buffer. Cell lysates and immunoprecipitates were analyzed on Western blots using mouse monoclonal anti-HA antibody (1∶800; Covance) (for exogenous 14-3-3ε) or anti-GFP antibody (1∶800; Sigma) (for exogenous Cdc25B) and anti-GAPDH antibody (1∶4000).

### Statistical analysis

Differences in GVBD rate of oocytes microinjected with different mRNAs were evaluated using the Chi-square test. Differences in the MPF activity assay were evaluated by one-way analysis of variance followed by a Least Significant Difference (LSD) test. A P value less than 0.05 indicates a significant difference.
